# Differing interpretations of health care encounters: A qualitative study of non-Latinx health care providers’ perceptions of Latinx patient behaviors

**DOI:** 10.1371/journal.pone.0236706

**Published:** 2020-08-06

**Authors:** Lucía I. Floríndez, Daniella C. Floríndez, Dominique H. Como, Rita Secola, Leah I. Stein Duker

**Affiliations:** 1 Mrs. T. H. Chan Division of Occupational Science and Occupational Therapy, University of Southern California, Los Angeles, CA, United States of America; 2 SOS Mentor, Los Angeles, CA, United States of America; 3 Hematology Oncology Service Line, Emergency Department, Children’s Hospital, Los Angeles, CA, United States of America; Massachusetts Department of Public Health, UNITED STATES

## Abstract

**Introduction:**

Due to provider shortages, it is probable that non-Latinx health care providers (HCPs) will treat Latinx patients. Because of this discrepancy, both providers and patients are likely to experience barriers and cultural differences during medical encounters. This article discusses select cultural factors and behaviors such as language, communication styles, and health care practices of Latinx families through the lens of their non-Latinx HCPs. The purpose of this study was to examine how non-Latinx HCPs perceive and describe certain behaviors they observe during healthcare visits with Latinx patients and families, and to illustrate how those behaviors can alternatively be interpreted as representing Latinx cultural norms.

**Methods:**

This qualitative study used a template coding approach to examine narrative interviews conducted with 18 non-Latinx HCPs to report how they described interactions with and the behaviors of their Latinx patients. Template codes were based on well-established Latinx cultural norms (e.g., *familismo*, *respeto*, *personalismo*, *simpatía*, *confianza*).

**Results:**

Many HCP descriptions of Latinx patient behaviors were coded into the Latinx cultural values categories (*familismo*, *personalismo*, *simpatía*, *respeto*, and *confianza)* by the research team. Results suggest that HCPs were not aware of how several of their patients’ behaviors may be culturally grounded, and that cultural differences between HCPs and their Latinx patients may exist.

**Discussion:**

Understanding how Latinx-specific cultural norms may be exhibited by Latinx patients and their families during healthcare encounters has potential to improve providers’ understanding of patient behavior, helping to promote culturally congruent care for Latinxs.

## Introduction

Currently, approximately 56 million Latinxs live in the United States, accounting for 18% of the total population and the second fastest growing minority population [1]. Though an increasing number of these Latinxs and Latinx Americans living in the United States speak English, one third of the Latinx population speak English “less than very well” and are considered limited English proficient (LEP) [1]. However, despite the surge in the US Latinx population, the number of Latinx physicians in the country has not followed the same trend. In 2010, there were only 105 Latinx doctors for every 100,000 Latinxs; in California, the state with the highest Latinx population, this number was only 50 physicians per 100,000 Latinxs [2]. Therefore, as the ratio of US Latinx patients to Latinx physicians continues to decline secondary to differences in growth trends, there is a high likelihood that non-Latinx healthcare providers (HCPs) will work with Latinx patients. Due to this discrepancy, both providers and patients are likely to encounter communication barriers. These include lack of professional interpreters or use of informal *ad-hoc* interpreters [3], non-translated or incorrectly translated health materials [4], and resources that are not tailored to be culturally appropriate or relevant [5–7].

In addition to communication challenges, racial/ethnic-related obstacles to health care exist for the Latinx population that can lead to health disparities. For example, Latinxs cite experiencing health care discrimination from providers [8, 9] and lack of access to helpful HCPs [10]. A sense of mistrust among Latinx patients for their providers [11–13] and provider bias toward their patients [14, 15] are also reported throughout the literature. These racial and ethnic-related barriers have the potential to influence receipt of quality health care [11, 16] as research suggests that Latinxs receive lower quality of care compared to non-Latinx white patients, as reported by patient self-report [17] and also consensus from a panel of experts on health disparities [18].

Beyond language and racial/ethnic barriers, Latinxs exhibit unique culturally-related needs that are not addressed by the current U.S. healthcare system which is designed to serve the non-Latinx white culture [17, 19, 20]. Culture is a shared set of values, beliefs, norms, practices, patterns of communication, familial roles, and other social regularities [21]. Although one should not assume homogeneity within the Latinx culture as it is diverse, encompasses many distinct ethnicities, and is influenced by national origin, nuances of language, and social class [22], the predominant influence of similar values create commonalities in the culture, reinforced by the shared language. Culturally sensitive research with Latinx populations is more than ethnic or language matching; rather, it is the “identification of and proper response to the needs and preferences of communities” [23].

Some of the shared cultural norms of U.S. Latinxs are *familismo*, *respeto*, *personalismo*, *simpatía*, *and confianza*. *Familismo*, the importance of immediate and extended family networks in the life of a person [24], means family is highly valued and a collective group orientation toward decision-making is adopted. *Respeto* embodies respect for one's elders and courtesy to others, including obedience to authority [25, 26]; this is exhibited in formal settings and requires respect for different positions of power, such as HCPs. *Personalismo* and *simpatía* refer to personal engagement and friendliness [27], and can be observed in interpersonal relationships. The stronger these relationships, the more *confianza*, or trust, exists between those involved. Together, these tenets reflect certain aspects of Latinx culture and create clear connections among Latinxs in the US [24, 28, 29]. The consideration of culture is a critical component in providing high-quality care to diverse communities and reducing racial and ethnic health disparities.

Given the well-documented difficulties Latinx patients experience while navigating a U.S. health system that is not culturally designed for them [30, 31], a traumatic medical situation such as a sudden health emergency or shocking medical diagnosis can be overwhelming. Of these, a child’s cancer diagnosis is devastating, impacting quality of life, social support, emotional strain, and family dynamics [32]. Among children in the United States, cancer is the leading cause of disease-related mortality [33]. Health care disparities for Latinxs extend to the field of pediatric oncology, with research suggesting that Latinx children, compared to their white counterparts, are less represented in cancer-related clinical trials [34], have poorer outcomes and receive more inconsistent care once diagnosed with cancer [32, 35], have elevated mortality risk [36, 37] and worse survival rates [38], and a higher likelihood of infection-related death [39]. Language barriers and immigration status have previously been associated with negative care experiences for Spanish-speaking caregivers of pediatric cancer patients, including receiving less diagnostic information from their providers [40], and feeling like they would have received better care if English were their primary language [41].

Little research has explored the Latinx pediatric cancer experience; those that have indicate a strong cultural relationship (including language, ethnicity, and cultural norms) between Latinx patients and how they perceive their care. For example, a review emphasized the importance of delivering culturally competent care for Latinx children with cancer and their families in order to improve the perceived quality of health care visits and prevent feelings of mistrust [42]. Culturally competent care “incorporates the importance of culture, assessment of cross-cultural relations, vigilance toward the dynamics that result from cultural differences, expansion of cultural knowledge, and adaptation of services to meet culturally unique needs” [43]. Munet-Vilaró [42] asserts that communicating with Latinx families of cancer patients needs to be done in a culturally appropriate way and consider elements of Latinx cultural norms.

Therefore, the purpose of this study was to examine how non-Latinx HCPs perceive and describe certain behaviors they observe during oncology healthcare visits with Latinx patients and families, and to illustrate how those behaviors can alternatively be interpreted as representing Latinx cultural norms. By critically examining these areas of cultural differences, we aim to advance cultural awareness and understanding of Latinx patient behavior and promote health equity.

## Methods

The data presented in this article report on a secondary analysis of a larger qualitative study aiming to explore the environmental barriers and facilitators to care for children with cancer receiving outpatient chemotherapy infusions. As the original research sought to describe experiences of patients and HCPs involved in outpatient chemotherapy infusion, a qualitative descriptive methodology was chosen to illustrate the details of these encounters and the meaning the participants attributed to their experiences [44]. Using this approach, the aim of the research was not to answer any a priori hypotheses or make assumptions about outpatient chemotherapy infusion. Instead, the focus was on collecting information via participant stories, and identifying and exploring themes that arose within and across interviews. Ethical approval was granted by the University of Southern California Health Sciences Institutional Review Board (15–00709) and the Hospital Review Board (15–00405). All participants provided oral consent, as suggested and approved by the Institutional Review Board, with verbal agreement documented in audio recordings. Excerpts of transcript data relevant to this study are available from the study PI (Stein Duker) or the first author (Floríndez) upon request.

### Participants

For the larger study, children with cancer undergoing outpatient chemotherapy infusions, their caregivers, and HCPs working in an outpatient Infusion Center with pediatric cancer patients were recruited. This study examines data only from the HCP group. Consecutive sampling of HCPs working in a single large urban pediatric hospital in California that treats a largely Latinx clientele (60–70%) who responded to brochures and flyers posted in the hospital and/or in-person recruitment presentations took place. HCPs who met inclusion criteria currently worked with pediatric oncology patients in the hospital outpatient Infusion Center. All types of providers were eligible (e.g., nurse, Child Life Specialist, physician’s assistant); our sample was primarily composed of nurses, as they represent the larger group of HCPs in the outpatient Infusion Center. Twenty HCPs met criteria and participated in the study. In the secondary analysis reported here, HCPs who self-identified as Latinx were excluded (n = 2); 18 participating non-Latinx providers were included in the data analysis for this manuscript (n = 18). Given the qualitative descriptive approach, a sample of 18 was considered large enough to capture rich descriptions of healthcare encounters and achieve broad context examples [45]. See Table 1 for a description of the sample.

**Table 1:** Descriptive characteristics of healthcare providers.

		Healthcare Provider (n = 18)
		Mean (SD)
**Age**		40.1 (11.2)
**Years of experience**		15.6 (10.6)
Years working at current hospital*		6.81 (5.86)
		**N (%)**
**Sex**		
	Male	1 (5.6)
	Female	17 (94.4)
Race*		
	White	7 (38.9)
	Asian	4 (22.2)
	African American	2 (11.1)
	Native American or other Pacific Islander	1 (5.6)
	More than one above	1 (5.6)
	Missing	3 (16.7)
**Hispanic Status**		
	Not Hispanic/Latinx	18 (100)
**Job Title**		
	Nurse	17 (94.4)
	Child Life Specialist	1 (5.6)
**Speaks Spanish (self-report)**		
	Yes	1 (5.6)
	Some	4 (22.2)
	Understand but not speak	1 (5.6)
	No	12 (66.7)

* Full demographic information not available for three providers.

### Data collection

Semi-structured interview questions were developed to elicit detailed stories about barriers and facilitators to positive chemotherapy infusion experiences, as reported by HCPs, caregivers of children with cancer, and children with cancer; only HCP perspectives are reported in this article. Questions were crafted by the authors and reviewed by experts in: qualitative research, pediatric oncology nursing, pediatric clinical psychology, and holistic symptom management. Questions included prompts about experiences providing infusions to children; perceptions of the challenges experienced during infusions; ideas regarding how to make the infusion experience better for all involved; collaborations with other HCPs; and if the provider spoke Spanish and whether or not that impacted their interactions with patients. Although a question list was used to guide the interview, the interviewer was instructed to further probe based on participants’ verbal and nonverbal responses as well as any other salient experiences that participants wished to discuss. Interviews were conducted by a bilingual (English/Spanish) member of the study team who identifies as Latinx; she has completed both didactic and in vivo interview training and has over twelve years of interview experience. Interviews took place in a private room or isolated areas within the hospital in order to maintain HCP privacy, lasted an average of 36 minutes each (range of 18 minutes to 79 minutes), and were digitally recorded and transcribed verbatim. Participants were provided with a $30 gift card.

### Data analysis

For the larger study, thematic analysis [46] was used to describe barriers and facilitators to positive outpatient chemotherapy infusion experiences, with a constant comparative method [47] utilized to analyze thematic similarities, differences, patterns, and relationships across the data. Two members of the research team independently read and coded four transcripts before meeting to create a provisional list of codes and sub-codes developed inductively from the data. Then, using the agreed upon list of codes, team members went back to all transcripts to independently code, using QSR International’s NVivo 11 qualitative data analysis software before meeting to compare coded materials.

As Latinx researchers, three of the authors (LF/DF/DC) have experience navigating the current health care system and encountering barriers with care providers related to cultural differences. Sensitized to such situations from their own lives, during the analytic process the coders observed subtle, nuanced themes related to the way the HCPs described their interactions with Latinx families. Influenced by their lived experiences, the coders were cognizant of how Latinx cultural norms could have influenced certain interactions described by the HCPs, and how the behaviors exhibited by the Latinx patients and their families were perhaps being misinterpreted by the HCPs. Two authors (LF/DF) then re-analyzed the data utilizing thematic analysis using a template coding approach [48]. Template codes were based on well-established Latinx cultural norms (*familismo*, *respeto*, *personalismo*, *simpatía*, *confianza*) and all transcripts were re-coded (LF/DF/DC) to examine how HCP descriptions of healthcare encounters might be related to these Latinx cultural values.

Qualitative researchers are encouraged to describe the lens from which they view the data, as well as how the researchers influenced the participants and vice versa [49]. Therefore, during the data analytic process, the authors engaged in regular discussion about how their own experiences and subjectivities influenced the process of analysis and kept notes on transcripts to document potential researcher biases; debriefing meetings with the larger research team also occurred at regular intervals. The few discrepancies in coding were resolved through discussion until a consensus was reached. Strategies to support the credibility and trustworthiness of these findings included: co-coding, peer researcher debriefing, maintaining an audit trail for analytic decisions, and critical analysis of potential author subjectivities and biases during the analytic process [50]. All 21 items of the Standards for Reporting Qualitative Research (SRQR) reporting guidelines were addressed [51].

## Results

Data suggests that non-Latinx HCPs may interpret behaviors that reflect commonly exhibited cultural norms (e.g., *familismo*, *personalismo*, *simpatía*, *respeto* and *confianza)* differently than Latinx families, possibly leading to misunderstandings and conflict. In this paper, scenarios described by HCPs were chosen by the research team in order to illustrate the cultural differences between the HCPs and their Latinx patients.

### *Familismo*: Families come in groups to infusions

As explained above, the presence and support offered by family members throughout life is highly valued by Latinxs [52]. However, several HCPs voiced concern with Latinx patients bringing multiple family members and/or extended family members to accompany them to chemotherapy infusions. Providers explained that although the hospital had regulations to prevent these occurrences, they were not enforced; staff felt these large groups caused disruptions due to small room size and increased volume of noise. For example, one nurse described that “…there's some Hispanic families that have eight kids and they bring all of them. Sometimes, mom and dad both come and all the siblings.” Bringing extended family members were also reported as problematic, exacerbating challenges, as one nurse described that

Sometimes families will come with extended family…it’s too crowded and too noisy, there’s too many people in the room… Like, grandma and grandpa might come with them… Or…it’s a school free day…and the four kids can’t be left at home so everyone comes.

Another nurse noted that

[Bringing the family] becomes a disruption. We try not to tell them—because if it’s one parent that comes with three kids you can’t leave the kids anywhere, so we know there’s no choice so we don’t usually say. But we do say, oh can you please not turn the TV loud.

These quotes illustrate the nurses’ characterization that extended families are noisy and loud, and the reason for sibling(s) attending an infusion is due a lack of other childcare options available. Some providers attributed the presence of these large groups to the economic difficulties of the Latinx families, suggesting that all children were brought because “…people who have socioeconomic issues may not have babysitters to hire and so you all have to come.” Likewise, another nurse reinforced this perception, stating that

They're the ones who bring in the husband and wife 'cause *nobody works* [emphasis added] and the four kids in addition to the kid who's getting treatment and they get put in small rooms… Those are the families that should get the big private rooms. However, why do they get the private room, just because they bring more people 'cause they can't afford childcare for whatever reason?

As applied to healthcare, understanding and appreciating *familismo* means that all family may wish to be present during health care visits, especially given the unexpected nature and high stress of cancer prognoses and care. One provider acknowledged the support system of the family, but also was quick to mention the disadvantages of having everyone present, explaining

There are cultures that definitely bring like everybody, and that’s their support system. They bring the whole support system, and that includes all the children and the aunts and the uncles and grandma and grandpa and everybody. And they don’t all fit in that room…but they try.

Again, it is clear that a lack of physical space limits the support of *familismo* and having the extended families present during visits. Furthermore, some HCPs struggled with the perceived fairness of accommodating large families over other families with fewer members at their visit.

### *Personalismo* and *simpatía*: Sharing and helping others

Multiple nurses who observed Latinx patients and their families described examples of the values of friendship and warmth (*personalismo)*, commenting that they often saw families share a sense of camaraderie in the clinic, for example

… I see the Hispanic families…Maybe because they can't speak like a lot of English. If they have two Hispanics in the same room the moms are very conversant and talking. And a lot of times the kids do play together.

In recounting stories about communication challenges for Latinx patients, HCP narratives often included descriptions of the help and support offered from other Latinx families. For example, when bilingual Latinx families helped interpret medical encounters for Latinx LEP families, “One parent will ask me [the nurse] something. We’ll be having a language issue. And the parent on the other side of the curtain will get involved because they speak English and Spanish. … [The family that doesn’t speak English] is appreciative.” In describing the stories in which the families were helpful to one another, the nurse is describing the Latinx cultural norms of *personalismo*, a tendency toward being warm and friendly toward others [67], and *simpatia* [53], a value promoting positive social interactions.

However, these interactions were also described as complicated, as they often blurred the lines of confidentiality. Simultaneously, HCPs expressed concern about the quality of translation and the accuracy of the information being delivered by the *ad-hoc* interpreter. Though HCPs recognized that having one family serve as an *ad-hoc* interpreter for another family was problematic, they also acknowledged it was often inevitable due to the close proximity of the families in shared rooms; additionally, providers underscored the inclination of Latinx families to want to help one another, despite not having formal training or education about medical issues. For example, one nurse stated that

[One family is interpreting for another family] And that is so inappropriate…But they’re just on the other side of a very thin sheet of fabric. So they already know what’s going on. They might as well get involved…Parents translating for each other sounds like, yeah, that’s like a workable thing. But it shouldn’t be that way. It’s not appropriate. Even though they want to help each other, and it seems like a natural like, yeah, yeah, I’ll just step in and help you out…They [a bilingual family in the room with the non-bilingual family] know what she’s saying, and they know that I don’t know what she’s saying…they want to help, and they’re not like, I’m going to intentionally violate their privacy…I don’t think that they see anything wrong with it. I see a lot of things wrong with it… Because I’m on like this—I’m in it, but I’m not—that interaction is not mine.

However, when an interpreter was not available and medical care needed to take place regardless of an interpreter being present, HCPs relied on a variety of people in the clinic to assist with communication; for example, “Most of the time people either use a coworker who might know Spanish, or the older child who might interpret. Sometimes they might use a different family. So, all those are not appropriate to do.” In the case of using children or siblings as interpreters, HCPs emphasized that

We are not supposed to put kids in that position, and we should not put kids in that position [to interpret for their family]. And I do not want to put kids in that position, but sometimes I have to put the kids in that position.

### *Respeto* and *confianza*: Families are grateful and won’t complain

*Respeto* and *confianza* were exemplified by encounters where nurses reported that Latinx patients and their families were subjected to seemingly worse care or conditions in the hospital, yet families did not complain about their uncomfortable situations. One nurse said,

[There are the donor] patients that always get the private rooms. And then you can get like the Hispanic family that doesn’t speak English and you know they're not going to complain, they just take what they get and they're happy to get treatment.”

Another HCP echoed the same sentiment, explaining that, “I have noticed that some of the, you know, like the Hispanic families, they don’t speak very good English…and they…don’t complain that much… they’re just okay in one [small room].” One HCP discussed the situation, saying, “I think this may be why they may feel discriminated against.”

Another HCP also explained her perception about how Latinx families are treated in the clinic, bringing up possible issues of unfairness,

So, maybe…I think [some families get] more space [than others]. There should just be more like equality maybe, because I think if you’re one of those parents or person that gets put in one of the little scrunched up spaces [rooms], like maybe that’s the thing that sets you off. I don’t know if it makes you feel discriminated against or what.

### Summary

In summary, one nurse unknowingly described an encounter that encompassed all of the above Latinx cultural tenets, where multiple large family groups (*familismo*) share a tiny space, don’t complain (*respeto* and *confianza*), and end up as friends (*personalismo* and *simpatia*), recounting that:

Other times too I will see this, you put those two Hispanic families—who you know aren’t going to complain—in the worst bed, you put them in the worst room with no light, worst curtain in the middle in the teeny tiny room even though they have all these family members. Well, they happen to both speak Spanish and be able to communicate with each other. So they are all a sudden best friends talking about their kids’ treatment, their kid has the same diagnosis, that is one opportunity for those guys to meet… They [the two different families] like definitely just kind of seem like, you know, become best friends in a day.

## Discussion

Understanding how culture influences health-related communication and decision-making among Latinx patients and their families is imperative to providing culturally competent care and addressing implicit bias among HCPs. Discussing the ways non-Latinx HCPs interpret their Latinx patients’ behaviors during healthcare encounters is a necessary first step to foster greater understanding. In this study, comments from non-Latinx HCPs suggest a lack of awareness of different Latinx cultural norms, in addition to revealing implicit bias held by some of the HCPs. Therefore, improving HCPs’ understanding of health encounters with their Latinx patients and families is the focus of the remainder of our paper.

### Familismo

When speaking about the presence of extended family, HCPs failed to situate family as a social support and illness as a shared burden in their Latinx patients [54]. Instead of displaying cultural awareness of *familismo*, many HCPs viewed the presence of large families as burdensome and problematic, and attributed the large familial presence to socioeconomic reasons (e.g., “nobody works”, unable to find/afford child care). These statements exemplify the HCPs’ lack of understanding of Latinx culture, as well as an underlying implicit bias toward their Latinx patients. Rather than encourage the extended support system of the Latinx family, the HCPs framed the family presence as negative and distracting, noting that the extra bodies made their job more difficult in a confined space. Interestingly, there is some truth to the description of Latinx patients’ economic plight, as Spanish-speaking caregivers reported higher rates of quitting their jobs when their child was diagnosed with cancer [41]. This is likely due to some Latinx’s holding jobs that do not allow for paid leave, such as migrant farm workers, landscapers, or household cleaning staff [55]. Indeed, in a study of Latinx migrant workers facing catastrophic illness, including cancer, their family members/caregivers were more likely to quit work entirely due to the stringent nature of the work (harvesting crops) and the lack of benefits [56].

When describing the extended family, nurses stated they are “too noisy” and that “the [family] is disruptive.” However, we counter that it is the physical space, too small to accommodate all family members, which is truly disruptive to the foundation of the Latinx culture’s social support network. Understandably, HCPs are concerned with the safety of performing infusions with multiple people and minimal space. However, we suggest the importance of being aware of the experience of Latinx families when they feel unwelcome and how this may impact the child’s health and/or quality of care; in this restricted space, Latinx culture is unfamiliar [4] leaving everyone with a difficult situation to navigate.

### *Personalismo* and *simpatía*

HCPs expressed discomfort when circumstances forced them to allow other families to help interpret for their patients in order to overcome language barriers. One HCP stated that “it is so inappropriate,” but was unable to stop it when one family extends the offer to help interpret for another family, stating “that interaction is not mine”, distancing herself from the conversation and relinquishing responsibility. The use of *ad-hoc* interpreters is in contradiction to the Department of Health and Human Services Cultural Linguistic Appropriate Services (CLAS) standards, which require free interpretation and translation services to be offered to LEP patients [57]. Although the hospital employees in this study receive annual training regarding this topic and guidelines to utilize approved interpretation services (e.g., language and cultural specialist staff, qualified bilingual employees, video and/or phone interpreting), it is imperative to remind and re-educate staff about these standards, despite the challenges enforcing implementation when patients and families are in close proximity to each other or when there may be urgent translation needs. *Ad-hoc* interpreting has proven problematic and unreliable in previous studies [23] in terms of the quality and breadth of substantive information that is being shared. Despite this, the discourse between patients and families is also representative of a vital part of communion, connectedness, and the need for positive social interactions among Latinx patients wanting to share their expertise and abilities to help others [58]. The HCPs’ narrative regarding this behavior is an un-nuanced analysis of the Latinx culture, indicating another area for greater improvement and cultural competency.

The communication barriers represented by families interpreting for each other and pediatric patients or siblings interpreting for their parents is also indicative of the larger problem that there is a severe lack of Latinx HCPs who can serve the growing Latinx population. Studies have shown that LEP patients have more problems understanding a medical situation than their English-speaking counterparts [59, 60], and Spanish-speaking patients report being dissatisfied with professional medical interpreting services [61]. Additionally, patients who do not speak the same language as their HCP risk receiving less than optimal care [62], and experience higher costs of care [63]. Previous research has indicated that minority patients have decreased trust in the health system in general, and express concerns about exploitation [34]. Other studies have implied that minority patients may be less likely than whites to be offered participation in clinical trials [64]. For all of these reasons, it is imperative to have HCPs that identify with their patients, both linguistically and culturally. However, despite 15 million Latinxs living in California [65], only 9 percent of enrollees accepted into medical schools in California are Latinx, and California ranks last in Latinx-doctor-to-patient ratio [66]. This dearth of Latinx HCPs directly impacts patients, who report feeling like they would receive better care if they spoke English [41].

### *Respeto* and *confianza*

In our data, HCPs describe the inherent bias of the health care system, explicating how some Latinx families have worse experiences, but how nothing is done to alleviate the situation because the Latinx patients “don’t complain”. These HCP reflections display a greater awareness of the plight of Latinx patients (e.g., Latinx families getting smaller and more undesirable rooms but not complaining); however, these anecdotes simultaneously present situations in which those patients are being taken advantage of. Recognizing that the system is lacking, but not working to change or address it, these providers play a tacit role in this disparity of care. These subtle instances of discrimination have the potential to alter the Latinx patient experience. As one HCP explained, “I think this may be why they may feel discriminated against.” She recognizes that in these small but cumulative actions, lies the root of, at worst, feeling discriminated against, and at best, recognizing you are being treated differently.

Based on results from a survey administered to English-speaking and Spanish-speaking LEP caregivers of pediatric cancer patients, Zamora et al. [41] found that LEP respondents were less aware about the details surrounding their child’s care plan and clinical trial enrollment, were less likely to call for information over the phone, were significantly more likely to quit their job in order to care for their child, and sometimes delayed medical treatments due to concern over their undocumented status. However, despite these difficulties, including waiting upwards of 90 minutes between checking in to the clinic and receiving chemotherapy treatment, Spanish-speaking caregivers reported higher satisfaction with their child’s care than their English-speaking counterparts, suggesting that Latinxs are happy with any care, regardless of quality [41]. Future research should examine Latinx patient perspectives of treatment as well as the impact awareness of perceived inequities may have on Latinx patients’ health outcomes.

In summary, it is apparent that Latinx patients and families have cultural values that are not adequately addressed or understood in the present health care system and the Latinx experience does not quite fit seamlessly into the traditional space, environment, or cultural norms of care. For English speaking patients, the medical system is structured to meet their needs, both language and social (e.g., chatting with a nurse in English, reading signs in English, completing paperwork in English). For a Latinx patient and family, not only is there evidence to suggest that not everything is translated properly, but we suggest that aspects of the medical environment such as lack of physical space, language barriers, and potential cultural misunderstandings make them a population that is easier to label as “other,” and therefore more likely to encounter discrimination. The HCPs are an example of the medical system at large, whose words illuminate the otherness that differentiates the Latinx experience from non-Latinx experience. Operating with limited resources and space, it is easy to understand why the Latinx patient experience stands out as different, why we point to these moments and mark them as “other”, rather than embracing and incorporating these values into the space. Understanding cultural differences and their influence on patient behavior is a crucial first step in developing culturally congruent care in order to eliminate situations which have the potential to create undue, extraneous burdens on the family and patients and negatively impact perceived sense of well-being and health outcomes. As explicated in this study, cultural misunderstandings exist in healthcare encounters; future work should focus on illuminating cultural differences and working to improve understanding in order to practice culturally congruent care.

## Limitations

This study was a secondary analysis of a larger qualitative study, which therefore led to multiple limitations. First, the data in this study included a small sample of HCPs who provided a specific type of care, so our results are not generalizable to all HCPs across service areas or outside of this geographic region, nor are we implying that all HCPs have the same values or beliefs. We interviewed HCPs about their current and past experiences; therefore, we were unable to confirm how participating HCPs knew their patients were Latinx and have no other information on those patient demographics, including acculturation levels or language preference. For this study, we relied on the fact that HCPs had access to the medical records of their patients to confirm Latinx identity; HCPs often described patients who spoke fluent Spanish as a proxy for assuming Latinx status. In addition, saturation for data collection was based on the goals of the parent study, not the sub-analysis presented here. However, with the data from our 18 participants, we were able to capture rich encounters of healthcare encounters that illustrated each of the template themes explored in this study. Lastly, as this line of research emerged inductively during the data analysis process, we were unable to confirm results using member-checking.

## Conclusion

Appreciating and recognizing the complex relationship between Latinx culture and health care is essential to understand how culture influences behavior and beliefs among Latinx patients. Cultural factors can only be adequately addressed when clinicians understand Latinx cultural themes which may be present during health visits. HCPs first need to appreciate that the interactions they observe may be grounded in Latinx culture; once aware, education about Latinx culture may help providers increase their understanding and comfort with Latinx patients, helping to reduce stereotypes and counter implicit bias. Strategies for HCPs include completing cultural competency education, enrolling in bias training programs, and maintaining CLAS standards when interacting with non-English speaking patients. Patients and their families may benefit from improved provider awareness of cultural norms, encountering greater acceptance of their actions and lessening perceptions of stigmatization. Additionally, LEP families may value culturally appropriate health resources published in their language of choice [67] as a way to further their health literacy and engagement in the health care system. Greater research, training, and cultural sensitizing is needed for all hospital staff and personnel to improve understanding of Latinx culture and reduce health disparities, as well as identifying resources to relieve structural space and environmental barriers.
